# Acceptability of Self‐Sampling for Cervical Screening in Ethnically Diverse Groups in Northwest England: A Focus Group Study

**DOI:** 10.1111/hex.70338

**Published:** 2025-07-31

**Authors:** Sophie Whitley, Rachel L. Hawkins, Jennifer C. Davies, Jiexin Cao, Lee Malcomson, Emma J. Crosbie, Lorna McWilliams

**Affiliations:** ^1^ Manchester Centre for Health Psychology, Division of Psychology & Mental Health, Faculty of Biology, Medicine and Health University of Manchester Manchester UK; ^2^ NIHR Manchester Biomedical Research Centre Manchester UK; ^3^ Division of Cancer Sciences, Faculty of Biology, Medicine and Health University of Manchester Manchester UK; ^4^ Department of Obstetrics and Gynaecology, St Mary's Hospital Manchester University NHS Foundation Trust, Manchester Academic Health Science Centre Manchester UK

**Keywords:** cervical screening, community‐based participatory research, ethnicity, prospective acceptability, self‐sampling, thematic framework analysis

## Abstract

**Introduction:**

Research indicates disproportionately low cervical screening uptake by diverse ethnic groups in England. If acceptable, self‐sampling might address population‐specific barriers and improve screening uptake. The Alternative CErvical Screening (ACES) Diversity study aimed to explore the prospective acceptability of self‐sampling (urine sampling and self‐swabbing), as an alternative to current cervical screening, among women from diverse ethnic groups.

**Methods:**

A qualitative study design was employed using focus groups. Forty‐eight women from diverse ethnic groups were recruited via community partners in Northwest England and a cross‐sectional survey. Eight focus groups were conducted (one online and seven in‐person; four with interpreters for Mandarin, Cantonese, Polish and Urdu). Data were transcribed, translated and analysed in English using thematic framework analysis guided by the Theoretical Framework of Acceptability.

**Results:**

Three themes were identified. ‘Cultural considerations’ explored how aspects of culture and faith influenced perceptions of self‐sampling. ‘Desire for comfort and control’ reflected views of how self‐sampling increases autonomy by maintaining privacy, potentially reducing both pain and tension associated with screening. ‘Confidence in testing’ illustrates beliefs about self‐sampling, around ease of use, practical challenges and accuracy concerns.

**Conclusions:**

Self‐sampling for cervical screening was considered highly acceptable. If introduced, self‐sampling could increase cervical screening uptake amongst women from diverse ethnic groups. Having a choice in how to interact with the screening programme and continuing to raise awareness of cervical screening were considered important. Future research should explore the concurrent or retrospective acceptability of urine self‐sampling for cervical screening.

**Patient or Public Contribution:**

Multiple public involvement discussion sessions in Northwest England‐based community centres were arranged with women to explore and build understanding about cervical screening and speak about the ACES Diversity study. A further session was held, with an interpreter, to discuss the focus group topic guide and study design with women and create an opportunity for any feedback. Written feedback was provided for the recruitment poster from seven women (two East Asian, two Central and Eastern European, two African‐Caribbean and one South Asian).

## Introduction

Primary high‐risk human papillomavirus (HPV) testing for cervical screening is expected to save lives [1]. In the United Kingdom, the NHS Cervical Screening Programme (NHSCSP) provides population‐based screening for cervical cancer using speculum‐based cervical sampling [2]. However, despite its benefits, participation is declining. Recent NHSCSP data reveal that whilst 80% coverage is a performance criterion, the percentage of eligible people within Greater Manchester up‐to‐date with their appointments is as low as 57.42%–78.51% [3]. Additionally, GP practices in income‐deprived areas face reduced screening rates [4]. Concerningly, women from diverse ethnic groups are significantly less likely to attend than their white British counterparts [5, 6].

Universal attendance barriers include low perceived risk and/or competing priorities, the absence of symptoms, fear and embarrassment and previous negative experiences of cancer screening or healthcare [7]. Barriers experienced by women from diverse ethnic groups include limited knowledge of cervical cancer or the purpose of screening, exacerbated by language‐related access issues and healthcare organisation differences from other countries [6, 8, 9]. Stigma, shame and misconceptions linking cervical cancer to sexual activity also play a role within communities [10].

Research shows the anticipated increase in global use of self‐sampling for cervical screening (‘self‐sampling’) [11]. Self‐sampling has been piloted for non‐attenders of the NHSCSP in London, demonstrating increased uptake, especially if self‐sampling was offered opportunistically [12]. Self‐sampling shows promising test efficacy [13, 14, 15] and cost‐effectiveness [16], which are key factors when considering changes to national screening programmes. A large‐scale UK study, HPValidate, revealed promising test accuracy for several self‐collection devices and HPV testing workflows when compared to speculum‐based screening [17]. Urine sampling, utilising a specialised first void urine collection device, the Colli‐Pee (Novosanis), has shown promising test accuracy to date [15, 18, 19, 20]. Further, self‐sampling methods are potentially more cost‐effective [16] and less carbon‐intensive [21] than current screening.

Public acceptability will be critical to integrating self‐sampling into the NHSCSP [22]. Research indicates its perceived benefits in convenience, privacy and reduced embarrassment [23, 24]. Quantitative data show high willingness among women from various ethnic backgrounds to use vaginal self‐swabs for cervical screening [25, 26]. However, studies have only recently commenced exploring the acceptability of urine sampling, primarily using questionnaires [27, 28]. Whilst the Colli‐Pee was considered concurrently acceptable to the women surveyed, ethnicity data were not reported [27]. A study by Drysdale et al. [28] found an overall preference for urine sampling (prospective acceptability) in women from diverse ethnic groups. However, it did not reference collection utilising the Colli‐Pee device, an imperative process for non‐inferior urine test accuracy [15]. Recent qualitative research revealed favourable views of vaginal swabbing and urine sampling for improving cervical screening uptake [29, 30], including for the Colli‐Pee [30]. However, further qualitative research focused on the detailed acceptability of self‐sampling across different methods is needed among diverse ethnic groups who may not speak English natively.

Other studies have captured views of diverse ethnic groups; however, they considered groups in isolation [31, 32]. Including multiple ethnic groups allows for comparisons across experiences, potentially revealing differing perspectives that can be factored into intervention development and evaluation [7]. In doing so, a contribution can be made to the national responsibility to make research more inclusive [33].

Ascertaining prospective acceptability of self‐sampling could be pivotal to improving cervical screening uptake of under‐screened women from diverse ethnic groups [25]. Acceptability refers to how appropriate an intervention feels, based on one's expected or actual cognitive and emotional responses [34]. The Theoretical Framework of Acceptability (TFA) was developed to assess healthcare interventions and comprises seven constructs: affective attitude, burden, perceived effectiveness, ethicality, intervention coherence, opportunity costs and self‐efficacy [34]. It has been applied to cervical screening, exploring perspectives of changing screening intervals [35]. Exploring facets of acceptability helps identify what aspects of an intervention may require refining during development and implementation [36].

This study aimed to explore the perspectives of women from diverse ethnic groups on the prospective acceptability of self‐sampling as an alternative to current cervical screening.

## Materials and Methods

### Design

A mixed‐methods design explored the acceptability of self‐sampling for women from diverse ethnic groups (named ACES Diversity) in Northwest England. The study consisted of two phases: (i) a cross‐sectional survey and (ii) qualitative focus groups. This paper reports on findings from Phase 2. Focus groups are commonly used in healthcare research to explore people's experiences and views by generating discussion [37]. They inform innovation by generating valuable feedback on novel health interventions [36, 38].

### Participants

Women were eligible to participate if they had a cervix and belonged to an ethnic group other than white British/Irish. Participants were invited to take part via established ACES Diversity community partners and through the survey.

### Procedure

Focus groups were held either in person at community centres (seven focus groups) or online (one focus group), between June 2023 and January 2024. Numerous public involvement sessions were held at community centres so that women could learn more about cervical screening and have a choice in providing feedback about the study. Women advised on places to advertise to facilitate recruitment, favoured video explanations of self‐sampling methods and, recommended holding focus groups at community centres. Written feedback for qualitative study materials was received.

Participants were recruited to in‐person focus groups with the support of community partners, who obtained contact details from interested women and provided participation information sheets. Documents were translated into the preferred language by an external translation service or verified by speakers with medical training. For the online focus group, women who completed the survey and agreed to be contacted for future research were invited. Purposeful sampling of women under age 35 was used following a review of sample demographics to incorporate younger women's perspectives.

All focus groups were co‐facilitated by two of three researchers (S.W., R.L.H. and L.Mc.W.), with one researcher leading. A semi‐structured approach was adopted to flexibly use probing questions to delve deeper into matters arising during data collection [39]. All women provided informed consent for participation and provided demographic details and self‐reported screening attendance (see Appendix A), either written (in‐person focus groups) or oral (online focus group). Oral consent was obtained using Zoom breakout rooms and audio recorded.

Focus groups were conducted in the groups' preferred language and audio‐recorded using two voice recorders. Language interpreters enabled the successful facilitation of diverse ethnic group participation [40]. Selected interpreters were women, mostly recruited via our ACES Diversity study community partners and often known to the women participating. Researchers met with all but one interpreter (obtained via NHS translation services) before the focus group, who were briefed on the research aims and objectives. For non‐English focus groups, the lead researcher spoke to the women, one sentence at a time, which was then translated. Interpreters were asked not to translate every spoken word into English. Field notes were taken firsthand by the second researcher or summarised from interpreters' notes.

A topic guide (see Appendix B) was developed through collaborative discussion between researchers, informed by TFA constructs and later revised following feedback. Women were shown one of two videos as a visual aid before answering questions specific to the acceptability of each self‐sampling method. Videos illustrated how to complete self‐sampling, using a Colli‐Pee ([41]; created by a study team member J.C.D.) or vaginal self‐swabbing [42]. Following each video, self‐sampling devices, the Colli‐Pee and FLOQswab (Copan), were handed out or shown [43].

Afterwards, women were thanked, offered voucher reimbursement and debriefed, offering reimbursements for childcare and travel costs. Reflexive notes were made. Recordings were transcribed verbatim by an external organisation; groups conducted in non‐English were translated into English during transcription. Three non‐English spoken focus groups, translated and transcribed by external organisations, were returned with some quotes unallocated to any participant. Three researchers (S.W., R.L.H. and L.Mc.W.) independently checked the transcripts, and supporting quotes were identified. Transcripts were sense‐checked by listening to the full audio recording and manually reviewing and editing any missed or incomplete speech and anonymised by replacing organisation names and applying pseudonyms (sensitive to participants' ethnicity). Confidentiality was protected through the use of anonymised data, separate storage of interview and personal data, and ensuring personal data was only accessible to researchers through a secure data storage drive. A minority of the dataset remained unidentifiable and was included in the analysis.

### Researcher Positioning

The first author (S.W.) is a white British female research assistant and former master's student. The other researchers are a white British PhD student and former research project coordinator (R.L.H.) and a white Scottish female research fellow (L.Mc.W.). All are involved in and highly supportive of cancer prevention and early detection research. The wider research team, with clinical and research expertise, supported engagement with community centres. For full researcher positioning, see Appendix C.

### Data Analysis

Data analysis was led by S.W. with support from R.L.H. and L.Mc.W. A TFA‐guided thematic framework analysis [34, 44] was used to explore the prospective acceptability of self‐sampling as an alternative to current screening. This approach is flexible and suited to team‐based analysis of large datasets [45]. The following stages were followed: transcription, familiarisation, coding, developing and applying a working analytic framework, charting, and data interpretation [45]. A critical realist stance was adopted [46]. Here, the research team acknowledges that study participants ascribed meaning to their nuanced perspectives according to their experiences and context, for example, their culture, gender identity and language. Their situated realities remained not fully accessible to researchers.

Coding was performed by S.W. and R.L.H. Both independently completed the inductive coding of one transcript and met to discuss similarities and discrepancies in coding. Then S.W. and R.L.H. double‐coded the same transcript on NVivo 14, annotating and adding new codes. Meanwhile, S.W. also analysed two familiar transcripts. Due to broadly similar consistency in coding, researchers coded the remaining transcripts independently and formed a draft framework, which was continually refined. Case classifications were created for each participant. To chart data, codes related to self‐sampling and based upon the draft framework, S.W. created matrices of all TFA constructs [34], with two additional matrices capturing remaining data related to the ‘future of screening’ and ‘delivery and design improvements of self‐sampling’. Matrices were exported to Excel, and the data within and across cases within each matrix and across matrices were considered to help uncover interrelated concepts and link shared meaning. Themes and sub‐themes were formed by grouping these patterns of data in a primarily inductive approach to prioritise women's perspectives, with reference to the relevant TFA constructs. Field notes and broader codes about screening helped contextualise women's experiences and views towards self‐sampling. The proposed thematic structure was reviewed and refined through collaborative discussion between researchers (S.W., R.L.H. and L.Mc.W.), with final structure unanimously agreed.

## Results

Forty‐eight women participated in one of eight focus groups (duration between 57 and 108 min), each with four to nine women. For participant demographics and prior screening attendance, see Table 1. To protect anonymity, ethnic groups have been broadened including to reflect continents.

**Table 1:** Sample demographics (*n* = 48).

Characteristic	Number of participants (%)
Age (years)	
20–29	6 (13)
30–39	9 (19)
40–49	13 (27)
50–59	9 (19)
60+	10 (21)
Unspecified	1 (2)
Gender	
Woman	48 (100)
Gender assignment same as birth gender	
Yes	48 (100)
Geographical or ethnic identity^a^	
South Asian (Bangladeshi, Indian, Nepali and Pakistani)	15 (31)
East Asian (Chinese and Hong Konger)	15 (31)
Black (African, Afro‐Caribbean, Black British and Black British Caribbean)	10 (21)
European (Greek and Polish)	5 (10)
Mixed	2 (4)
Prefer not to say	1 (2)
Religion	
Christian	17 (35)
Muslim	14 (29)
No religion	12 (25)
Hindu	2 (4)
Other	2 (4)
Prefer not to say	1 (2)
Employment status^b^	
Unemployed	10 (20)
Self‐employed	9 (19)
Employed part‐time	8 (17)
Employed full‐time	7 (15)
Full‐time carer/Homemaker	3 (6)
Unable to work	4 (8)
Retired	7 (15)
Student	2 (4)
Disability	
No disability	34 (71)
Physical disability (Including sensory impairment)	4 (8)
Another experience of disability	4 (8)
Prefer not to say	3 (6)
Unspecified	3 (6)
Learning disability (including developmental disorders)	0 (0)
Previous attendance at a cervical screening appointment	
Yes	37 (77)
No	10 (20)
Unspecified	1 (2)
Index of multiple deprivation	
1st–2nd Decile	21 (44)
3rd–4th Decile	9 (19)
5th–6th Decile	1 (2)
7th–8th Decile	5 (10)
9th–10th Decile	2 (4)
Unable to calculate	9 (19)

^a^ Groupings made to protect the identity of participants.

^b^ Multiple participants selected multiple categories of employment status, chosen to be represented separately.

Most women reported screening attendance; however, barriers to screening were prominently discussed, as reported in Table 2. In total, three themes were produced: (1) cultural considerations, (2) desire for comfort and control and (3) confidence in testing, with women's experiences of screening and healthcare more broadly providing additional context.

**Table 2:** Barriers to current cervical screening identified across focus groups.

Barriers to current cervical screening^a^
Association of testing with sex
Cultural taboo
Discomfort
Embarrassment
Fear
Fear of virginity loss
Gender of the healthcare professional
Lack of awareness that screening exists
Lack of confidence to attend
Lack of education
Lack of hygiene
Lack of information
Lack of resources
Language
Low perceived need for screening
Marital status
Menstruation
Modesty
Other healthcare professional‐related barriers
Pain
Privacy—no conversations with others
Resistance to change away from the current speculum screening due to older age
Scheduling of an appointment
Sexual inactivity
Spousal disapproval
Trauma from prior bad experiences
Unable to have a trusted person present during screening
Unsuitable provisions to accommodate other health conditions

^a^ In alphabetical order.

### Theme 1: Cultural Considerations

Women across most focus groups discussed how aspects of their culture and faith influenced views of screening and self‐sampling. Aspects ranged from marital status, modesty, hygiene and cleanliness, to wider considerations about inequality, low awareness of screening, and screening in other countries.

Despite acknowledging the advantages of not exposing oneself to others, self‐swabbing was perceived as inappropriate for some, especially by Muslim women. Similarly to current screening, self‐swabbing could be morally compromising, ‘*But you won't go through with this [self‐swab] test if you're a virgin…. They won't even request it. It's just for us [who are married]*’ (Ade, Black, FG4). Others agreed with Ade about self‐swabbing being inappropriate for unmarried virgin women, ‘*That's what I'm saying*’ (Naila, South Asian, FG4). In comparison, urine sampling was favoured and viewed as accessible regardless of marital status. Similar views were apparent in focus groups without Muslim women. Many highlighted apprehensions about exposing themselves during screening, with modesty considered an important societal expectation for some: ‘*…our culture is more conservative, especially down there, it's very private. So, in our culture, despite me having been here for so long, I still feel embarrassed*’ (Qing, East Asian, FG7). Considering this, urine sampling might be preferred by women due to its discreetness, especially if the Colli‐Pee is used for other medical tests:

‘This [Colli‐Pee] is very, it's not invasive, or anything, and it's not offensive […] rather than just being, oh this is to do with sex, and wombs and so it can actually be normalised, if you use something like this for standard urine samples.’

Kimona, Black, FG6

The importance of normalising tests was echoed by Brigitte, with the example of a Covid test used, ‘*Well, imagine, the COVID test wasn't normalised, but it is now…. And this could, like she said, be introduced in society, as normal…*’ (Brigitte, Black, FG6).

Personal hygiene was another important consideration for some women, with cleanliness emphasised as an integral element of female Muslim culture, ‘*Because when I go [to screening], I just make sure I'm clean…. But everybody is different. That's us, it's our culture. That's a cultural thing, I think, cleaning is what we do…*’ (Uzma, South Asian, FG2). Other Muslim women discussed how cleanliness could be less of a concern when using self‐sampling methods, ‘*You need to make sure that you need to be clean, whatever, so there is an extra effort in there. And with these self‐sufficient methods, you don't need that, so quite a lot of positives then*’ (Daria, South Asian, FG2).

Strong views were held about how one size does not fit all, including how current UK healthcare fails to meet the needs of women from diverse ethnic groups. Women emphasised low cervical screening awareness among their communities, and strategies to improve this must be widely introduced as a priority in conjunction with any changes to sampling methods: ‘*We've got the Equality and Diversity Act, but really and truly, it's not covering the NHS*’ (Brigitte, Black, FG6). Additionally, women's prior experiences of screening abroad influenced perceptions of the NHSCSP and self‐sampling, with some receiving more frequent screening elsewhere or receiving screening at a younger age. The introduction of self‐sampling was viewed as an opportunity to make additional changes to the NHSCSP, such as more frequent testing: *I would like to know by using the new methods, will the interval become shorter that we do not need to wait to get tested every 3 years?* (Unidentified, East Asian, FG1).

### Theme 2: Desire for Comfort and Control

Feeling comfortable during screening was viewed as important. Women conceptualised comfort as encompassing both physical and emotional aspects related to privacy, pain and ‘relaxation’. Self‐sampling was considered to achieve greater autonomy and control in screening.

### Privacy

In contrast to current screening, participants across focus groups indicated that self‐sampling offers greater privacy, enabling women to participate independently and removing the need to rely on healthcare professionals ‘…*in the comfort of your own home, your own bathroom by yourself’* (Aisha, South Asian, FG2). However, one focus group highlighted that the privacy offered by self‐swabbing would be lost if assistance is required, ‘*Well, there on the video it said, if you need help. So, they obviously, it's like asking another person to help me…. There's no privacy, really, for this one*’ (Rahima, South Asian, FG4). Ade agreed, ‘*There's no privacy then*’ (Ade, Black, FG4).

Self‐sampling was discussed as less invasive than current screening, especially for urine sampling in comparison to self‐swabbing, ‘*Urine is obviously easier because it's not intrusive but I wouldn't be averse to that [self‐swabbing] one*’ (Nadia, South Asian, FG2).

### Pain(less) and Tension

Fear and uncertainty around potential pain from current speculum‐based screening were discussed, including by Hua, who was not yet age‐eligible for cervical screening ‘*I've never attended this kind of scan before, so I'm quite curious about the process. Will it hurt?*’ (Hua, East Asian, FG1). Whilst self‐sampling was viewed as likely less painful than current screening, many women perceived the non‐penetrative method as pain‐free. For Meilin, this reduced the fear associated with the current speculum examination, ‘*It is not painful. Just go to the toilet and urinate. It is very good. You do not need to be afraid of pain anymore*’ (Meilin, East Asian, FG7).

The self‐swab method appeared to promote autonomy and dignity in screening for several women. Participants highlighted benefits of having greater personal control over the pace and depth of sampling, ‘*But I don't know, it's like you said, right, it feels like it's what the nurse or doctor would do, except you're doing it to yourself so you can control the pace of it. I feel like that would be much less painful because I would be more relaxed*’ (Diya, South Asian, FG8). Whilst a few women considered self‐swabbing to be more comfortable than urine sampling, varying opinions were present. Some women regarded self‐swabbing as too invasive and ‘alien’ whilst others felt deterred due to the feeling of self‐swab materials, ‘*You know some people don't like the feel of cotton buds and stuff like that? Maybe it's a sensory or a texture thing, I just wouldn't use that…*’ (Maya, South Asian, FG2).

### Theme 3: Confidence in Testing

Across focus groups, women varied in confidence in ‘doing’ self‐sampling, yet also had wider concerns about the accuracy of self‐sampling. The perceived burden of ‘doing’ the self‐sampling was discussed in relation to physical health. Prior experiences of similar devices also underpinned self‐efficacy in completing self‐sampling.

### Ease of Use

None of the women had heard of self‐sampling for cervical screening before, and many asked related questions. Previous experiences of screening influenced acceptability towards self‐sampling methods, ‘*I don't mind going in [for current cervical screening] but I do feel like if there were easier ways, I'd probably opt for them more*’ (Sade, Black, FG8). Overall, many women regarded self‐sampling as more convenient, particularly if employed or studying full‐time, with no appointment needed and saving health service resources, ‘*I think the biggest advantage is it saves a lot of time, no matter for doctors, nurses, and patients ourselves*…’ (Mei, East Asian, FG1).

Most women discussed feeling confident about performing urine sampling, with the process considered to be easy, uncomplicated, user‐friendly, and similar to pregnancy tests. When comparing both self‐sampling methods, fewer women perceived self‐swabbing as either easy or involving a similar amount of effort to urine sampling. For these women, confidence had grown from using similar, familiar tests or items such as menstruation products or sexual health kits. Repeated use was expected to improve confidence further, as shown by a non‐attender's view, ‘*I think the people who will be using it for the first time, they might not feel as confident as people who have used similar options previously. But I think once they've used it, they might become confident for the next time*’ (Daria, South Asian, FG2).

Self‐sampling was perceived to potentially increase screening uptake, including for those never screened, ‘*I think these could [holding the colli‐pee] also be good for people who wouldn't…Like me, for example. Somebody that wouldn't go for an actual screening, but you still want to get tested…*’ (Tahira, South Asian, FG4). Many expressed personal willingness to complete self‐sampling, in contrast to current screening. Across focus groups, women had a strong preference for choice in opting for self‐sampling, the self‐sampling method, and how to receive the kit or return the collected sample, ‘*Yeah, that choice should be there, it should be open, it should be what do you want to do? Do you want to come in and have it done? Or shall we give you that? I think these two choices, both of these and the appointment with your doctors, I think that should be there, the choice should be there*’ (Uzma, South Asian, FG2).

### Practical Challenges

Various practical concerns about urine sampling were discussed. For example, providing an inadequate sample, not drinking enough water and holding urine for enough time, especially mid or post‐menopause, or during sickness. Dexterity issues and general health were viewed as making self‐sampling more difficult, ‘*When I was younger, slimmer, healthy in terms of skeletal system, this [self‐sampling] would not have been an issue for me. At the moment though, in my current health condition, I would definitely not undertake to complete this by myself, definitely not*’ (Maja, European, FG3). Kinga shared a similar viewpoint, ‘*That is true. There is a line there, where this needs to be put in, so people who are obese or ill … they won't be able to see that line, they will have a problem to bend over, move around*’ (Kinga, European, FG3).

Additionally, one woman described resistance to try new forms of urine testing, ‘*I prefer the bottle business…Yeah. I think it's because I'm so old…. Yeah, I'm set in my ways…. So, I don't want to change, I think that's what it is, really*’ (Nia, Black, FG5). Further, motivational factors impacted the likelihood of using self‐taken tests for one regular screening attender, suggesting self‐sampling might not be for all ‘*…Because my attitude is, if it's a self‐applied thing, it's not going to be done*’ (Maddie, Unknown ethnicity, FG5). Maddie's view generated varied opinions among others present, with one woman in agreement, ‘*Yeah, I had mine for a year before I done it*’ (Aaliyah, Black, FG5), whilst another disagreed, ‘*No, I done mine straightaway, because I'm concerned*’ (Destiny, Black, FG5).

For others, completing self‐testing incorrectly, especially the self‐swab, was a concern, ‘…*It's hard to see when it's right, do you know what I mean? [Self‐swab] won't even let you know if you've done it properly*’ (Naila, South Asian, FG4). Women felt uncertain about how far to insert the self‐swab or where to take the sample from and feared dropping the self‐swab. When similar doubts arose about completing urine sampling correctly at first go, some suggested a spare Colli‐Pee might help grow confidence, ‘*…I guess if there will be more than one sample, then maybe my confidence will increase and then I would be like, oh, what if the first one did not get on, I'll try another one next time when I go for a wee, yes*’ (Muna, South Asian, FG8). However, some criticised the sustainability of such tests made of non‐biodegradable plastic, and the additional resources used:

‘*Yeah, because if that's the final, if there's anything, they find anything, you have to go back anyway. There's no point, I think it's a waste of money, a waste of the doctor's time, a waste of the laboratory's time, and money…. That's true, there's a lot of plastic there.’*

Destiny, Black, FG5

### Test Accuracy Concerns

Many women expressed concerns about self‐sampling accuracy compared to current screening, which is generally viewed as a ‘gold standard’, completely reliable test. Accuracy worries were highlighted during all discussions about urine sampling, due to its perceived lack of ‘directness’: ‘*Because with the insertion of the speculum, or whatever they use, what they're getting from the neck of the womb, would they get it from urine, would they get the same thing?*’ (Aaliyah, Black, FG5). For some, low accuracy could be a deterrent to self‐sampling and women instead might opt for current screening, even perhaps at the cost of pain, ‘*I am thinking, I am not sure if I missed it, but has it been mentioned about [urine sampling] accuracy rate? I know it is definitely not 100%, but how high is its rate? If the check if not accurate, then it may be necessary to do the traditional test, which comes at a chance of being painful*’ (Ya‐ting, East Asian, FG7).

Double‐testing was considered important, ‘*I would do this test and then go and double‐check it, just in case*’ (Julia, European, FG3), especially among those with reported health conditions who expressed greater distrust of test result reliability due to previous experiences. This was exemplified by Alicja's perspective as the discussion continued: ‘*Double‐checking is always a needed thing. With every condition. One test is not enough if they find something, God forbid should something go wrong*’ (Alicja, European, FG3). Others questioned whether cross‐contamination whilst completing self‐swabbing might affect results: ‘*Because urine comes from inside of you…. Whereas with self‐swab, even though it goes inside, if the cotton bud is in contact with the air or bacterial, it might undergo some changes*…’ (Chun, East Asian, FG1). Resultantly, evidence that self‐sampling tests are as accurate as current cervical screening, inclusive of all ethnicities, was considered important, *‘And to make sure that it works with all nationalities, rather than just the European model, because it could be different with an Asian woman, could be different with a Chinese woman, it could be different with a Black woman. They just have to get it right, so we can catch everybody’* (Kimona, Black, FG6).

Most women thought it made sense to attend an in‐person follow‐up if self‐sampling was HPV positive, ‘*I would prefer using one of these rather than going to my GP for a smear test. But if it came back abnormal, then I would obviously go to the GP, and … […] And if, heaven forbid, if I found that, you know, it came back abnormal, then I would go back to the GP, I would make an appointment. And go through the channels, the necessary channels, definitely*’ (Laila, Mixed, FG6). However, when subsequently considering the reality of a self‐sample detecting HPV, women evaluated potential consequences. Self‐sampling could delay access to healthcare appointments and possible treatment if speculum‐based tests are required, ‘… *what I would be concerned about would be how hard [sic] can I make an appointment after this? How long will it take? Because I mean, cancer is something that's […] How do you say it? It's easy to get to the next stage. So, if the time period of waiting is actually long, I'm not sure if I'd rather do this by myself, or just go to the doctor*’ (Na, East Asian FG7).

## Discussion

This study explored prospective acceptability of urine sampling, collected with a novel first void urine collection device, and vaginal self‐sampling as an alternative to current cervical screening, gathering views from UK women from diverse ethnic groups. Overall, self‐sampling methods were acceptable, and views indicated a perceived willingness to use them, consistent with previous research [24, 27, 30, 47]. Previous qualitative studies have held focus groups to discuss both self‐sampling methods [29, 30]. However, this study extends this by applying the TFA [34] to explore prospective acceptability. Additionally, the present study conducted focus groups with non‐English‐speaking women, enabling a greater understanding of the perspectives of women from diverse ethnic groups. This, in turn, offers an in‐depth insight into the acceptability of urine self‐sampling, which has not yet been achieved in research.

Convenience appears important for increasing screening uptake. Women from diverse ethnic backgrounds commonly face time‐related barriers to current screening to a greater extent than white British women [48]. Moreover, self‐sampling has the potential to improve screening uptake in under‐screened women by overcoming general barriers [49, 50]. Women favoured comfort and privacy associated with self‐sampling [51]. Concerns about pain were often rooted in traumatic experiences of current screening, which can reduce screening attendance [6, 52]. In this study, self‐sampling was viewed positively, as more hygienic and less painful than the current screening.

Upholding virginity before marriage is important to women from multiple ethnic groups [53, 54]. As discussed, and shown elsewhere, attending current screening can lead to doubt among the community about virginity status if seen by others [55]. This extended to women's views of using the self‐swab only, whereas urine sampling was considered usable for virgin women. Additionally, South Asian women explicitly cited preferences for self‐sampling linked to cleanliness reasons. Cleanliness forms an integral part of Islamic faith and might partially explain this, as women do not need to present outward appearances to anyone else [56]. Preserving modesty also reduces embarrassment and offers discreetness when completing self‐sampling, echoing recent findings [30].

Accuracy concerns of self‐sampling tests were common, attributable to the novelty and perceived accuracy of the current screening. Women showed low confidence in their perceived capability of completing self‐sampling, which is in line with previous research [57]. Concerns mostly focused on women's health problems or processes impacting self‐sampling capability because of physical and/or functional limitations. Findings newly revealed fewer concerns about urine sampling than self‐swabbing, suggesting urine sampling or current screening might feel less burdensome for some.

Autonomy is considered important, and whilst most women favoured self‐sampling, women thought self‐sampling should not fully replace current screening. Maximising choice might lead to improved cervical screening uptake. A recent systematic review found higher screening uptake using directly mailed HPV self‐sampling kits compared with current screening, including for women from diverse ethnic groups [58]. Other studies have acknowledged the need for choice in screening methods, where most women would seemingly opt for self‐sampling with a preference for urine tests [28, 29, 59]. Therefore, the future implementation of self‐sampling within the NHSCSP should consider the choice of screening method.

Across focus groups, women advocated for greater awareness about cervical screening, and widespread advertising of self‐sampling methods if introduced. It remains of high importance to target communities like the South Asian community, address the highly prevalent issue of cultural taboo and reduce inequity in cervical screening [60, 61].

### Strengths and Limitations

A notable strength is the inclusion of underserved populations, most of whom resided in low socio‐economic areas within the United Kingdom. These populations have consistently shown reduced participation in the NHSCSP [4, 6] and are often excluded from health and care research [62]. Partnering with local community organisations and involving women at each stage ensured the study kept its relevance and meaning [63]. In doing so, it supports the goal of eliminating cervical cancer through equitable action [64].

Steps to improve accessibility included translating materials into requested languages, using interpreters, offering childcare and travel reimbursement, and avoiding scheduling during key religious dates. These efforts align with best practices for inclusive research with diverse communities ([65]; NIHR, 2023).

Yet, most researchers, including co‐facilitators, were white British women. We aimed to conduct research with cultural sensitivity, remaining attentive to women's needs and wishes, exploring perspectives with curiosity and without judgement. However, most researchers lacked personal cultural insight into the everyday challenges that women from diverse ethnic groups face [66]. Therefore, co‐facilitators felt hesitant to inquire about differences in screening uptake based on ethnicity, and this might have affected participants' comfort in discussing some topics. Although reflexivity was important, intersectionality remained largely undiscussed and could have yielded further insight. Intersectionality is a relatively underutilised lens of understanding in research about engagement with cancer care, especially for women from diverse ethnic groups [67].

Using the TFA [34] helped explore constructs of acceptability and manage the large dataset. However, the early stages of theme generation based on aligning with TFA constructs felt restrictive. Ultimately, the chosen approach captured data across matrices in both a meaningful and comprehensive manner. Further, certain TFA constructs held a greater weight of importance pre‐intervention. For example, perceived effectiveness was focused on test accuracy; however, it was not directly asked about. Despite the removal of an original TFA construct of ‘intention’ [34], explicitly uncovering willingness to attend a follow‐up test if high‐risk HPV‐positive remained important.

Another limitation was holding focus groups in languages other than English, in which the co‐facilitators did not speak. Having bilingual researchers has benefits; however, it remains unattainable for all studies, and the cultural competency of researchers is considered more important [65]. Although interpreters were briefed comprehensively, the co‐facilitator had less control over question phrasing. After the first focus group, back translation in real time was deemed unnecessary. This saves time, avoids interrupting the flow of discussions [68] and reduces interpreter burden. However, it reduces the opportunity for probing questions. Poor initial transcription quality also meant some participants remained unidentified despite revisions.

Several weaknesses relate to sample demographics. Collecting demographic details about migration status, type of generation and English language proficiency would have been useful. Low English language proficiency and being a first‐generation migrant are associated with reduced cervical screening uptake [69, 70]. Alternatively, women reported being mostly regular cervical screening attenders, perhaps opting to participate because they view cervical screening more favourably than others [31]. Obtaining a detailed cervical screening history would have strengthened the interpretation of findings. More efforts are needed to target recruitment of non‐attenders [6], to gain valuable perspectives.

### Implications for Practice and Future Research

Findings suggest the acceptability of self‐sampling across diverse ethnic groups, indicating the potential to reduce disparities in cervical screening uptake. Views about self‐sampling were explored with women from multiple diverse ethnic groups [32], helping to generate rich data. Findings provide initial support towards the implementation of self‐sampling in terms of acceptability. Future research should explore the experienced acceptability of self‐sampling among women from diverse ethnic groups to indicate how perspectives persist or shift over time [34].

Both urine sampling via Colli‐Pee and vaginal self‐swabbing show high sensitivity and are comparable to standard methods [15, 17]. Providing clear information about the high accuracy of both self‐sampling methods, if introduced, would offer women reassurance. Further, continuing to raise awareness of cervical screening and highlighting the availability of screening methods are strongly encouraged. Options suggested by women include community discussions with a healthcare professional and an interpreter present, improving advertisement (via TV, posters, word of mouth and self‐sampling collection points, e.g., GP practices and pharmacies), and healthcare practitioners discussing cervical screening at routine healthcare appointments. Video demonstrations and comprehensive, verbal explanations of self‐sampling methods were valued. Future recommendations include videos in different languages, and for the urine sampling video to include an animation of a woman collecting a sample.

## Conclusion

Findings show high prospective acceptability of self‐sampling methods, including both urine sampling and vaginal self‐swabbing, among women from multiple diverse ethnic groups. Views supported having a choice in how to interact with the screening programme. Coinciding with raising awareness for cervical screening, self‐sampling offers great potential to increase uptake of cervical screening amongst women from diverse ethnic groups. Future research should explore the experienced acceptability of self‐swabbing, especially urine sampling for cervical screening within these populations, in observational studies.

## Author Contributions

**Sophie Whitley:** methodology, investigation, project administration, resources, data curation, formal analysis, visualisation, validation, writing – original draft, writing – review and editing. **Rachel L. Hawkins:** conceptualisation, funding acquisition, methodology, investigation, project administration, resources, data curation, formal analysis, visualisation, validation, writing – review and editing. **Jennifer C. Davies:** conceptualisation, funding acquisition, methodology, resources, writing – review and editing. **Jiexin Cao:** project administration, investigation, resources, writing – review and editing. **Lee Malcomson:** funding acquisition, investigation, project administration, resources, data curation, writing – review and editing. **Emma J. Crosbie:** conceptualisation, funding acquisition, methodology, supervision, writing – review and editing. **Lorna McWilliams:** conceptualisation, funding acquisition, methodology, investigation, project administration, resources, data curation, formal analysis, visualisation, validation, supervision, writing – review and editing.

## Disclosure

The views expressed are those of the authors and not necessarily those of the NIHR or the Department of Health and Social Care. The funding sources had no role in the design of this study and will not have any role during its execution, analyses, interpretation of the data, or decision to submit results.

## Ethics Statement

The Proportionate University Research Ethics Committee at the University of Manchester granted study approval (REF: 2024‐15182‐32475).

## Consent

All participants were asked to read a participant information sheet before the focus groups, with copies available upon request at the focus groups if needed. All participants provided informed consent. For in‐person focus groups, participants provided informed written consent. For the online focus group, informed consent of each participant was audio‐recorded within a Zoom breakout room before conducting the focus group, in a separate file from the focus group recording.

## Consent for Publication

Participants consented to the publication of anonymised quotes. The identity of participants has been protected further by pseudonyms and groupings made for ethnicity.

## Conflicts of Interest

The authors declare no conflicts of interest.

## Data Availability

The data that support the findings of this study are available from the corresponding author upon reasonable request.

## Appendix A

**Participant Demographic Questionnaire**

**Participant reference number:**

**□ Request for information:** permission sought/participant agreed to provide the information below

**How old are you?** ______ years **What is your home postcode?** _________________

**How would you describe your gender? (please tick one):**

**□** Woman

**□** Man

**□** Non‐binary (neither man nor woman)

**□** Would rather not say

**□** In another way (please say: _______________________)

**Is your gender the same as the gender you were assigned at birth? (please tick)**

**□** Yes

**□** No

**□** Would rather not say

**Are you (please tick)**

**□** Employed full‐time

**□** Employed part‐time

**□** Self‐employed

**□** Unemployed

**□** Full‐time carer/homemaker

**□** Unable to work

**□** Retired

**□** Student

**Have you previously attended a cervical screening (smear) appointment (please tick)?** There are no right or wrong answers

**□** Yes

**□** No

**□** Unsure

**How would you describe your ethnicity (please tick)**

**□** White British or English, Welsh, Scottish or Northern Irish

**□** White Irish

**□** Gypsy or Irish Traveller

**□** Roma

**□** Any other white background (please specify):

**□** White and Black Caribbean

**□** White and Black African

**□** White and Asian

**□** Any other mixed/multiple ethnic background (please specify) _____________________________________

**□** Indian

**□** Pakistani

**□** Bangladesh

**□** Chinese

**□** Any other Asian background (please specify) ___________________

**□** African

**□** Caribbean

**□** Any other Black/African/Black British/Caribbean background (please specify) ______________________________

**□** Arab

**□** Any other ethnic group (please specify) ________________________

**□** Prefer not to say

**How would you describe your religion (please tick)**

**□** No religion

**□** Christian

**□** Buddhist

**□** Hindu

**□** Jewish

**□** Muslim

**□** Sikh

**□** Any other religion Please specify ________________________

**Disability: Which of the following describes how you think of yourself (please tick)**

**□** I do not consider myself to be disabled

**□** Physical disability (including sensory impairment)

**□** Learning disability (including development disorders)

**□** Another experience of disability

**□** Prefer not to say

## Appendix B Focus Group Topic Guide

**Current cervical screening**

– Before we start to talk about possible new methods of cervical screening, I first want to hear your views about the current way cervical screening is offered to women.
– First of all, what interested you in coming to this group today to talk about cervical screening?
– Can you/anyone tell me how they feel about the current cervical screening (sometimes called a smear test)? What do you think about it? [If it seems like they are unsure what cervical screening is, ask them if they know what it is and then briefly explain depending on response(s); PROMPTS: knowledge of Human Papillomavirus HPV]
– Are there any barriers that might stop you or have stopped you from accessing cervical screening? When we say barrier, we mean something that prevents a person from attending or accessing cervical screening.
  - Explore barriers: What might make that a barrier? How to overcome this barrier?
– Data/Research says that uptake of cervical screening is lower in some ethnic groups; what are your thoughts about this?
  - Prompts, for example, are they the same for all communities? Are there things that are important to accompany this data (e.g., numbers might be lower, but this is because information about screening when invited is not provided in a language other than English; you have to find it)?
  - Prompts: if conversation directs towards wider NHS/systemic issues experienced, does or could this have an impact on cervical screening experiences/attendance?
I will now share with you two short videos (one at a time) that describe two self‐sampling methods [share screen on video call/email videos to participant], the vaginal self‐swab and the urine test. I also have some written information about these methods, which may help you to think about these methods during the interview [pass around for in‐person interview/focus group/email the information for telephone/share screen on video call to show videos]

VIDEOS [*Researcher to describe the two self‐sampling methods that will be talked about during interview/focus group: Colli‐Pee device and vaginal self‐swab/use the visual aids and refer participants to the self‐sampling leaflet. Brief explanation about how if the self‐sampling test is HPV positive—would then need to book and follow up with cervical screening led by clinician (i.e., smear test)*]. You may find it useful to refer back to this information whilst I ask the next questions.

**Self‐sampling as an alternative method for the current cervical screening**

– **What are your first thoughts and reactions about self‐sampling for cervical screening?**
– **How would you feel about being offered this for cervical screening?**
– Specific discussion about Colli‐Pee as a method
  - Do you like or dislike the Colli‐Pee? [Prompts: positives/negatives of Colli‐Pee for cervical screening]
  - Is this a method of cervical screening that you would be comfortable following?
  - How confident would you be using this method? Why?
  - How much effort would it take to use the Colli‐Pee? [Prompts: how easy/difficult to use]
  - Any concerns about using this method?
  [If participants seem unsure, provide suggestions about what may make the test hard to do, e.g., it might be easier if you are able to complete the test at home, but it might be hard to know if you are completing the test correctly without a doctor being there. Explore how to overcome issues like that, e.g., provision of online demonstration videos within materials supplied for the test]
– Specific discussion about vaginal self‐swab as a method
  - Do you like or dislike the vaginal self‐swab? [Prompts: positives/negatives of Colli‐Pee for cervical screening]
  - Is this a method of cervical screening that you would be comfortable following?
  - How confident would you be using this method? Why?
  - How much effort would it take to use the vaginal self‐swab?
  - Any concerns about using this method?
– Will either or both these methods make you more or less likely to undertake cervical screening? Follow‐up questions are required to explain the reasons behind the answer.
– Do these methods help to get rid of any barriers to current cervical screening?
  [Prompt: If any cultural or religious elements of cervical screening or overall access to healthcare are discussed in the interview/focus group, e.g, *earlier you mentioned X is important/a barrier to you, would introducing self‐sampling change this at all?*
– Should self‐sampling (vaginal self‐swab and urine test) replace current cervical screening or should it be an option? [PROMPT: any preference for one over the other?]

If you tested HPV positive on the self‐sampling test, you would then need to go for an in‐person cervical screening sample taken by a doctor or nurse. What are your thoughts about this? Tell me more about how willing you would be to do this? [*Researcher may need to explain HPV]*

Overall, we are trying to understand the acceptability of self‐sampling methods for cervical screening. How acceptable are self‐sampling methods to you? [Prompt: do you think they are a good or bad idea?]

**Conclusion**

We have covered everything we hoped to, but is there anything that you would like to add that we haven't mentioned or that you thought we would discuss and haven't?

## Appendix C

**Full Researcher Positioning**

The first author (A) was a white British middle‐class female master's student, subsequently employed as a research assistant to continue the project. At the time of the research, A was not yet of an age eligible to receive a cervical screening invitation. Having received the HPV vaccination at school as part of the national HPV vaccine programme was positively regarded by A. The other focus group facilitators were a white Scottish female research fellow (C) and a white British research project coordinator (B), both largely involved in and highly supportive of cancer prevention and early detection research. Positive views were held by all types of population‐level screening programmes, as well as the promotion of informed choice in attending these, whilst being cognisant of their importance in early cancer detection, especially for underserved populations.

Other members of the research team included D, Professor of Gynaecology Oncology, white British; E, Speciality Trainee in Obstetrics and Gynaecology and Clinical Research Fellow undertaking a PhD in urine sampling for cervical screening, white Welsh; and F, Speciality Trainee in Obstetrics and Gynaecology and Clinical Research Fellow undertaking a PhD exploring urine sampling for cervical screening, Chinese. D, E and F are leading a research programme exploring the test accuracy and acceptability of urine sampling for cervical screening within the wider population, whose work within urine self‐sampling led to this study idea and the inclusion of the Colli‐Pee device for urine sampling within this study. These individuals held positive views of self‐sampling methods for cervical screening. Their work primarily focused on the [anonymised research study title and acronym], but they also undertook some of the initial focus groups and engagement with community centres, informed study design and materials, for example, Colli‐Pee, helped create accurate materials for the study groups and helped to advertise the present study. One such item was the topic guide, in which one extra area addressed was whether women were willing to undergo speculum‐based cervical screening if high‐risk HPV positive on a self‐sampling test. If unwilling, completing self‐sampling would defeat the purpose of this method of cervical screening, and therefore, it was a valid concern for the clinicians. Focus group facilitators were mindful about how their colleagues' passion for related work, testing the accuracy of these methods, contributed to their own initial appraisal of self‐sampling being positive. This might have had an influence on data analysis by being drawn to more favourable opinions on self‐sampling.

Early positive interactions between researchers and both community partners and interpreters contributed to the formation of mutual trust and shared understanding. However, the research team were predominantly educated, white British women, which likely negatively impacts all stages of the research. Intentional thought about having an insider‐outsider status was perceived to be essential [71]. Focus group facilitators were individually identified as both an insider (i.e., as a woman) and an outsider (i.e., not from a diverse ethnic group). To minimise this bias as much as possible, feedback from the PPI work was prioritised over the research team's views whenever possible. A is cognisant of her stance as a novice researcher and has shown concern at times over misrepresenting the views of women from diverse ethnic groups, given her own ethnicity. Therefore, we strove to consistently interweave women's own voices throughout this study. Reflective notes were kept throughout the research process, including after each focus group. This helped to challenge any held assumptions and analyse data referring to ethnicity or culture with added caution and sensitivity.
